# Chitosan-based intelligent theragnosis nanocomposites enable pH-sensitive drug release with MR-guided imaging for cancer therapy

**DOI:** 10.1186/1556-276X-8-467

**Published:** 2013-11-08

**Authors:** Eun-Kyung Lim, Warayuth Sajomsang, Yuna Choi, Eunji Jang, Hwunjae Lee, Byunghoon Kang, Eunjung Kim, Seungjoo Haam, Jin-Suck Suh, Sang Jeon Chung, Yong-Min Huh

**Affiliations:** 1 Department of Radiology, College of Medicine, Yonsei University, Seoul 120-752, South Korea; 2 YUHS-KRIBB Medical Convergence Research Institute, Seoul 120-752, South Korea; 3 Nanodelivery System Laboratory (NDS), National Nanotechnology Center (NANOTEC), National Science and Technology Development Agency (NSTDA), Thailand Science Park, Pathumthani 12120, Thailand; 4 BioNanotechnology Research Center, KRIBB, Yuseong, Daejeon 305-806, Republic of Korea; 5 Department of Chemical and Biomolecular Engineering, Yonsei University, Seoul 120-749, South Korea; 6 Department of Chemistry, Dongguk University, Seoul 100-715, South Korea

**Keywords:** Theragnosis, Cancer therapy, Drug delivery, pH-sensitive, Magnetic resonance imaging, Nanocomposites

## Abstract

Smart drug delivery systems that are triggered by environmental conditions have been developed to enhance cancer therapeutic efficacy while limiting unwanted effects. Because cancer exhibits abnormally high local acidities compared to normal tissues (pH 7.4) due to Warburg effects, pH-sensitive systems have been researched for effective cancer therapy. Chitosan-based intelligent theragnosis nanocomposites, *N*-naphthyl-*O*-dimethymaleoyl chitosan-based drug-loaded magnetic nanoparticles (*N*Chitosan-DMNPs), were developed in this study. *N*Chitosan-DMNPs are capable of pH-sensitive drug release with MR-guided images because doxorubicin (DOX) and magnetic nanocrystals (MNCs) are encapsulated into the designed *N*-naphthyl-*O*-dimethymaleoyl chitosan (*N*-nap-*O*-MalCS). This system exhibits rapid DOX release as acidity increases, high stability under high pH conditions, and sufficient capacity for diagnosing and monitoring therapeutic responses. These results demonstrate that *N*Chitosan-DMNPs have potential as theragnosis nanocomposites for effective cancer therapy.

## Background

Many therapeutic anticancer drugs are limited in their clinical applications because of their toxicities and low solubility in aqueous media [1-14]. For instance, doxorubicin (DOX) is one of the most widely used drugs in cancer therapy. However, it can cause side effects such as cardiotoxicity and drug resistance. Also, it is difficult to administer intravenously because of its low solubility in aqueous media. Nanomaterial-based drug delivery systems have received attention in overcoming this drawback. These systems can be made from a variety of organic and inorganic materials including non-degradable and biodegradable polymers, and inorganic nanocrystals. Polymeric micelles based on amphiphilic block copolymers have the advantages of high biocompatibility and drug-loading capacity with low toxicity because they can self-assemble into polymeric micelles in aqueous media [8,15-17]. They accumulate in tumors through an enhanced permeation and retention (EPR) effect compared to single small molecules, leading to preferential spatio-distribution in the tumor. However, the drug release behavior of polymeric micelles is difficult to control; they freely release the drug before reaching tumors, which could give rise to unwanted side effects and low therapeutic efficacy [4,8]. Well-designed drug delivery systems need to be developed to enable cancer chemotherapy that fundamentally enhances therapeutic efficacy by minimizing drug release in undesirable sites. With these systems, a precise drug concentration can be delivered to tumors to reduce side effects. Drug delivery systems can be designed to release drugs triggered by environmental parameters such as pH, enzymes, and temperature [16,18-29]. The pH-sensitive systems are of special interest because tumors and intracellular endosomal/lysomal compartments exhibit abnormally high local acidities compared to healthy tissues with a normal physiological pH of 7.4 [9,21,25,28-43].

**Figure 1:**
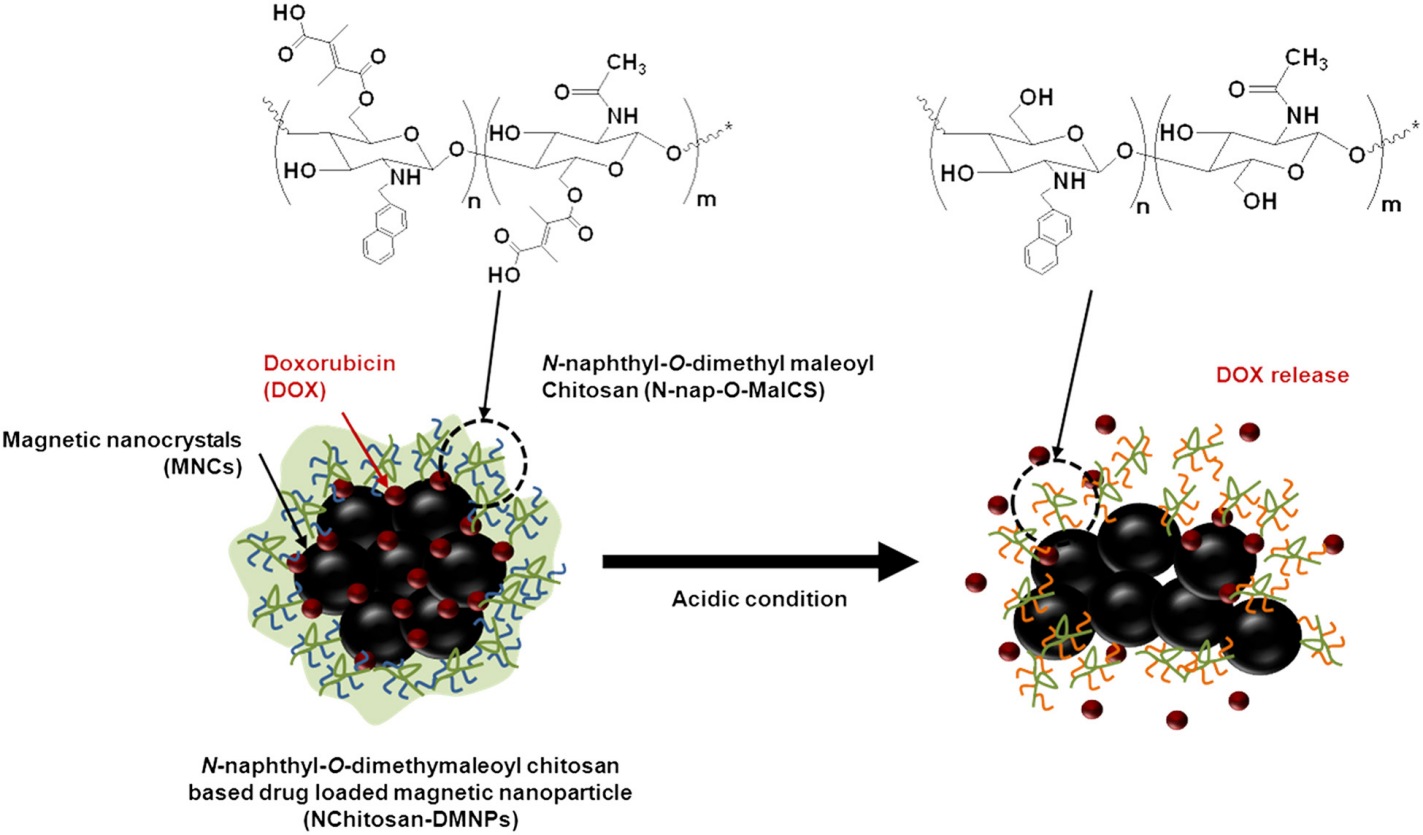
**Schematic illustration of *N*** **Chitosan-DMNPs enabling pH-sensitive drug release and MR monitoring for cancer therapy.**

In this study, chitosan-based intelligent theragnosis nanocomposites that enable pH-sensitive drug release with magnetic resonance (MR)-guided images were developed (Figure 1). This nanocomposite was based on *N*-naphthyl-*O*-dimethymaleoyl chitosan (*N*-nap-*O*-MalCS), a newly synthesized, pH-sensitive amphiphilic copolymer modified by maleoyl groups on a chitosan backbone. Chitosan is non-toxic, biodegradable, and non-immunogenic [44-72]. It is a linear polysaccharide consisting of *N*-acetyl-glucosamine (acetylated) and glucosamine (deacetylated) repeating units, and its abundant reactive groups facilitate chemical modification of functional groups. Hydrophobic magnetic nanocrystals were loaded as imaging agents in this system, leading to the formulation of theragnosis nanocomposites capable of delivery therapy concomitant with monitoring. This nanocomposite will allow effective cancer therapy because it can provide patient-specific drug administration strategies that consider drug-release patterns and biodistribution.

## Methods

### Materials

Chitosan with an average molecular weight (mol. wt.) of 15 kDa was purchased from Seafresh Industry Public Co., Ltd. (Bangkok, Thailand). The degree of chitosan deacetylation (DDA) was determined by ^1^H-NMR spectroscopy to be 98%. Cellulose microcrystalline power, chitosan with low molecular weight, 2-naphthaldehyde, 2,3-dimethylmaleic anhydride, sodium borohydride, sodium hydroxide (NaOH), triethylamine, *N*,*N*-dimethylformamide (DMF), dimethylsulfoxide (DMSO), *N*-hydroxysulfosuccinicimide (NHS), iron(III) acetylacetonate, manganese(II) acetylacetonate, 1,2-hexadecanediol, dodecanoic acid, dodecylamine, benzyl ether, paraformaldehyde, triethylamine, 2,3-dimethylmaleic anhydride, and DOX were purchased from Sigma-Aldrich (St. Louis, MO, USA). Ethanol and chloroform (CF) were obtained from Duksan Pure Chemicals Co. (Seonggok-dong, Danwon-gu, South Korea). Dialysis tubing with a molecular weight cutoff of 3,500 g/mol was purchased from Cellu Sep T4, Membrane Filtration Products, Inc. (Segiun, TX, USA). Phosphate buffered saline (PBS; 10 mM, pH 7.4) and Dulbecco’s modified eagle medium (DMEM) were purchased from Gibco (Life Technologies Corp., Carlsbad, CA, USA). All other chemicals and reagents were of analytical grade.

### Synthesis of ***N***-naphthyl-***O***-dimethylmaleoyl chitosan

**Figure 2:**
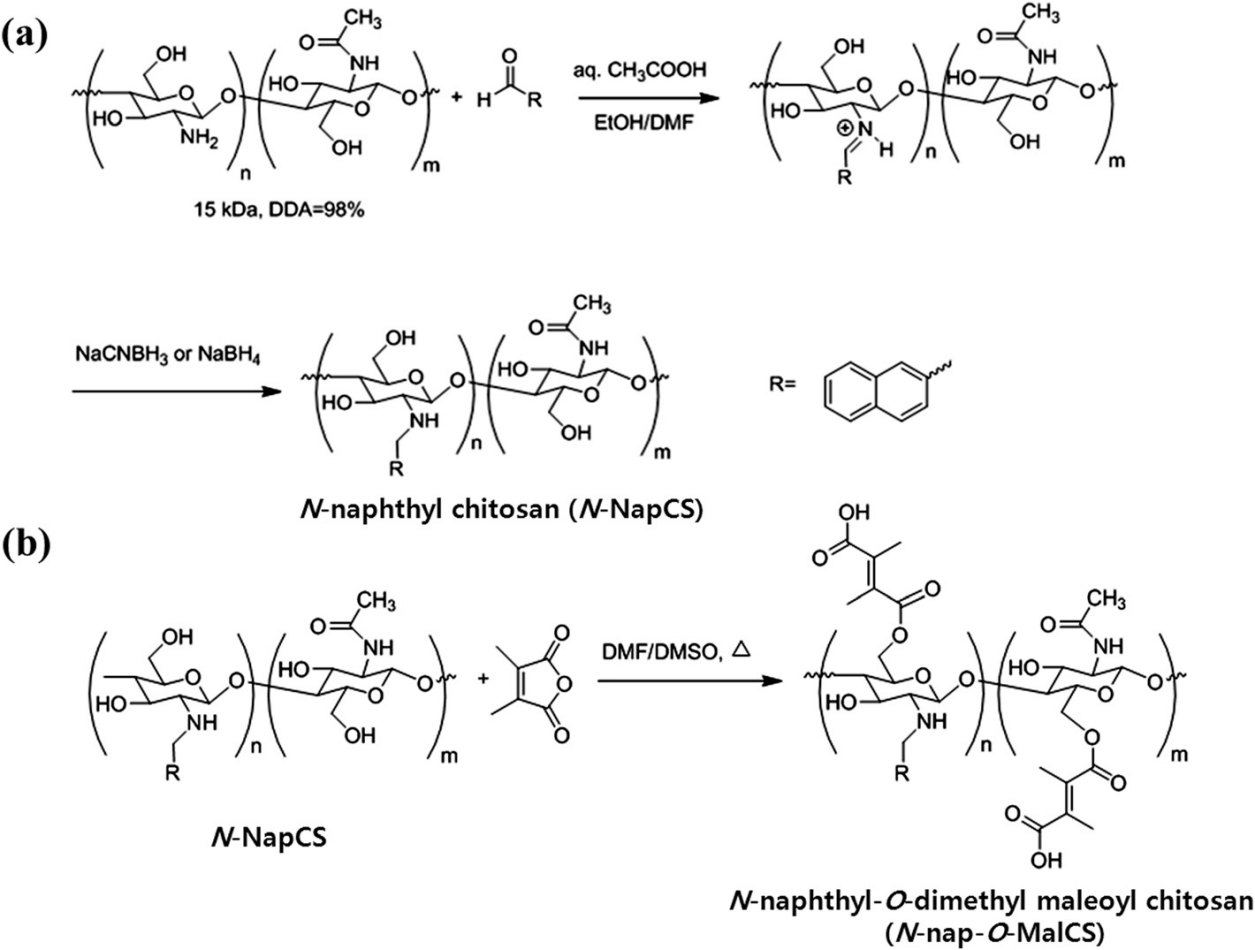
**Synthesis of (a) *N*-NapCS and (b) *N*-naphthyl-*O*-dimethylmaleoyl chitosan (*N*-nap-*O*-MalCS).**

*N*-naphthyl chitosan (*N*-NapCS) was synthesized by reductive amination (Figure 2a) [68]. Briefly, 1.00 g of chitosan (6.17 meq/GlcN) was dissolved in 50 mL of 1% (*v*/*v*) acetic acid (pH 4). 2-Naphthaldehyde (1.31 mL, 2.0 meq/*N*-NapCS) dissolved in 30 mL of DMF was then added and stirred at room temperature for 24 h. Solution pH was adjusted to 5 with 15% (*w*/*v*) NaOH. Subsequently, 3.50 g of sodium borohydride (15 meq/*N*-NapCS) was added and stirred at room temperature for 24 h, followed by pH adjustment to 7 with 15% (*w*/*v*) NaOH. The precipitate was collected by filtration and re-dispersed in ethanol several times to remove excess aldehyde. The precipitate was then filtered, washed with ethanol, and dried under vacuum. White *N*-NapCS powder was obtained (1.78 g). Each *N*-NapCS (0.50 g) was dispersed in 30 mL of DMF/DMSO (1:1 *v*/*v*). Triethylamine with the amount of 1 mL and 1.50 g of 2,3-dimethylmaleic anhydride were added. The reaction was performed at 100°C under argon purge for 24 h (Figure 2b). The reaction mixture was cooled to room temperature and filtered to remove insoluble residue. The mixture was dialyzed with distilled water for 3 days to remove excess 2,3-dimethylmaleic anhydride and solvent. It was then freeze-dried at -85°C under vacuum conditions for 24 h. A brown *N*-nap-*O*-MalCS powder was obtained (0.58 g).

#### ***Preparation of nanopolymeric micelles***

*N*-Nap-*O*-MalCS (12 mg) was dissolved in 12 mL of DMSO. The solution was stirred at room temperature until completely dissolved. It was then placed into a dialysis bag and dialyzed against deionized water overnight. The solution was then filtered through syringe filter membranes (cellulose acetate) with pore sizes of 0.45 μm for further study. Using the same procedure described above, the solution was then placed into a dialysis bag and dialyzed against deionized water by adjusting to pH of 8 to 9 with 5% (*w*/*v*) sodium hydroxide overnight.

#### ***Effect of pH and temperature on nanopolymeric micelles***

Three milliliters of nanopolymeric micelles was placed into a dialysis bag and dialyzed against 12 mL of PBS buffer of pH 5.5, 6.0, 6.5, 6.8, 7.2, 7.4, and 8.0 at 25 and 37°C for 24 h. PBS buffer was refreshed twice. The particle sizes of nanopolymeric micelles with different pH values were analyzed in triplicate by laser scattering.

#### ***Preparation of magnetic nanocrystals***

Monodispersed magnetic nanocrystals that are soluble in non-polar organic solvents were synthesized by thermal decomposition, as previously described [73-78]. Briefly, iron(III) acetylacetonate (2 mmol), manganese(II) acetylacetonate (1 mmol), 1,2-hexadecanediol (10 mmol), dodecanoic acid (6 mmol), and dodecylamine (6 mmol) were dissolved in benzyl ether (20 mL) under an ambient nitrogen atmosphere. The mixture was then preheated to 200°C for 2 h and refluxed at 300°C for 30 min. After reactants cooled down at room temperature, the products were purified with excess pure ethanol. Approximately 12 nm of magnetic nanocrystals (MNCs) were synthesized by seed-mediated growth method.

#### ***Preparation of N-naphthyl-O-dimethymaleoyl chitosan-based drug-loaded magnetic nanoparticles***

*N*-naphthyl-*O*-dimethymaleoyl chitosan-based drug-loaded magnetic nanoparticles (*N*Chitosan-DMNPs) were fabricated by nanoemulsion methods. Fifty milligrams of MNCs and 2 mg DOX were dissolved in 4 mL chloroform (CF). This mixture was then poured into 50 mL of pH 9.8 solution containing *N*-nap-*O*-MalCS (40 mg). The solution was ultrasonicated for 30 min and stirred overnight at room temperature to evaporate the CF. The resulting suspension was centrifuged three times for 15 min at 13,000 rpm. After the supernatant was removed, the precipitated *N*Chitosan-DMNPs were re-dispersed in 5 mL of deionized water. The size distribution and zeta potential of *N*Chitosan-DMNPs were analyzed by laser scattering (ELS-Z; Otsuka Electronics, Hirakata, Osaka, Japan). The loading ratio (%) and crystallinities of MNCs at 25°C were determined by thermogravimetric analysis (SDT-Q600, TA Instruments, New Castle, DE, USA) and X-ray diffraction (X-ray diffractometer Ultima3; Rigaku Corporation, Tokyo, Japan), respectively. The magnetic properties of *N*Chitosan-DMNPs were also analyzed using vibration sample magnetometer (VSM) (model 7407, Lake Shore Cryotonics Inc, Westerville, Columbus, OH, USA) at 25°C. The surface compositions were measured using X-ray photoelectron spectrometry (ESCALAB 250 XPS spectrometer; Thermo Fisher Scientific, Hudson, NH, USA).

#### ***Determination of drug release profile***

One milliliter of the above *N*Chitosan-DMNPs was centrifuged for 45 min at 20,000 rpm, and the precipitated *N*Chitosan-DMNPs were re-dispersed in 1 mL of buffer solutions at pH 5.5, 7.4, and 9.8. The dispersed particles were sealed in dialysis tubing and immersed in 10 mL of each buffer solution at 37.5°C, which was conducted in triplicate. The amount of released drug was measured at 593 nm by fluorescence spectrometry. These results are shown as average ± standard deviation (*n* = 3). In addition, the drug loading efficiency (7.2 wt.%) was measured in the same manner. Briefly, *N*Chitosan-DMNPs’ weight was measured after lyophilization and then dissolved in 1 mL of DMSO. The loaded amount of drug was measured by fluorescence spectrometry, using the following formula:

$$\begin{array}{c} \text{Drug loading effiency}\;\left(\mathrm{wt}.\%\right)\\ \;=\left(\text{Weight of drug in}\;N\text{Chitosan‒DMNPs}\right)/\\ \;\left(\mathrm{Weight of}\;N\mathrm{Chitosan‒DMNPs}\right)\times 100 \end{array}$$

#### ***Cellular internalization of NChitosan-DMNPs***

MR imaging and fluorescence microscopy confirmed cellular internalization of *N*Chitosan-DMNPs. NIH3T6.7 cells were obtained from American Type Culture Collection. First, these cells were seeded at a density of 1.0 × 10^6^ cells/well in six wells for growth overnight at 37°C and then further incubated with *N*Chitosan-DMNPs in 5% CO_2_ for 24 h at 37°C. The cells were washed three times with PBS and stained by Hoechst (Molecular Probes TM, OR, USA) to show nucleus location. Fluorescence microscopic images were obtained using a laser scanning confocal microscope (LSM700, Carl Zeiss, Jena, Germany). Under the same conditions, NIH3T6.7 cells treated with *N*Chitosan-DMNPs were washed twice, collected, and then re-suspended in 0.2 mL of 4% paraformaldehyde for MR imaging analysis. All experiments were conducted in triplicate.

#### ***Determination of cell viability using MTT assay***

The cell viability of *N*Chitosan-DMNPs was evaluated by measuring cell growth inhibition using a 3-(4,5-dimethylthiazol-2-yl)-2,5-diphenyltetrazolium bromide (MTT) assay (Roche Molecular Biochemicals, Mannheim, Germany) compared to DOX as a control. NIH3T6.7 cells (1.0 × 10^4^ cells/well) were implanted in a 96-microwell plate with temperature at 37°C overnight and treated with various concentrations of *N*Chitosan-DMNPs. After 24 h, the cells were washed and incubated for an additional 48 h. The yellow tetrazolium salt of MTT solution was reduced to purple formazan crystals in metabolically active cells. The cell viability was determined from the ratio of treated cells to non-treated control cells. The results are shown as average ± standard deviation (*n* = 4).

#### ***Animal experiments***

All animal experiments were conducted with approval from the Association for Assessment and Accreditation of Laboratory Animal Care (AAALAC) International. Tumor-bearing mice were developed, and NIH3T6.7 cells (5 × 10^6^ cells suspended in 50 μL saline per animal) were implanted into the proximal thighs of female BALB/nude mice (4 to 5 weeks of age) to investigate *N*Chitosan-DMNPs’ distribution and tumor growth rate. After tumor volume reached approximately 40 mm^3^ at 3 days post-implantation (0 days), *in vivo* magnetic resonance imaging (MRI) experiments were performed using *N*Chitosan-DMNPs (five mice). Comparative therapeutic efficacy was evaluated using three groups (saline, doxorubicin, and *N*Chitosan-DMNP) of mice (ten mice per each group). Animals were treated with equivalent doses of DOX (3 mg/kg) and *N*Chitosan-DMNPs suspended in PBS by intravenous injection every 2 days for 12 days. At predetermined time periods, the length of the minor axis (2a) and major axis (2b) of each tumor was measured using a caliper. Each tumor volume was then calculated using the formula for ellipsoid [(4/3)π × a^2^b].

#### ***MR imaging***

*In vivo* MR imaging experiments were performed using a 3.0 T clinical MRI instrument with a micro-47 surface coil (Intera; Philips Medical Systems, Best, The Netherlands). The *T*2 weights of nude mice injected with nanoparticles were measured by Carr-Purcell-Meiboom-Gill sequence at room temperature with the following parameters: TR = 10 s, echoes = 32 with 12 ms even echo space, number of acquisitions = 1, point resolution = 156 × 156 μm, and section thickness = 0.6 mm. For *T*2-weighted MR imaging in the nude mouse model, the following parameters were adopted: resolution = 234 × 234 μm^2^, section thickness = 2.0 mm, TE = 60 ms, TR = 4,000 ms, and number of acquisitions = 1.

## Results and discussion

### Characterization of ***N***-naphthyl-***O***-dimethymaleoyl chitosan

**Figure 3:**
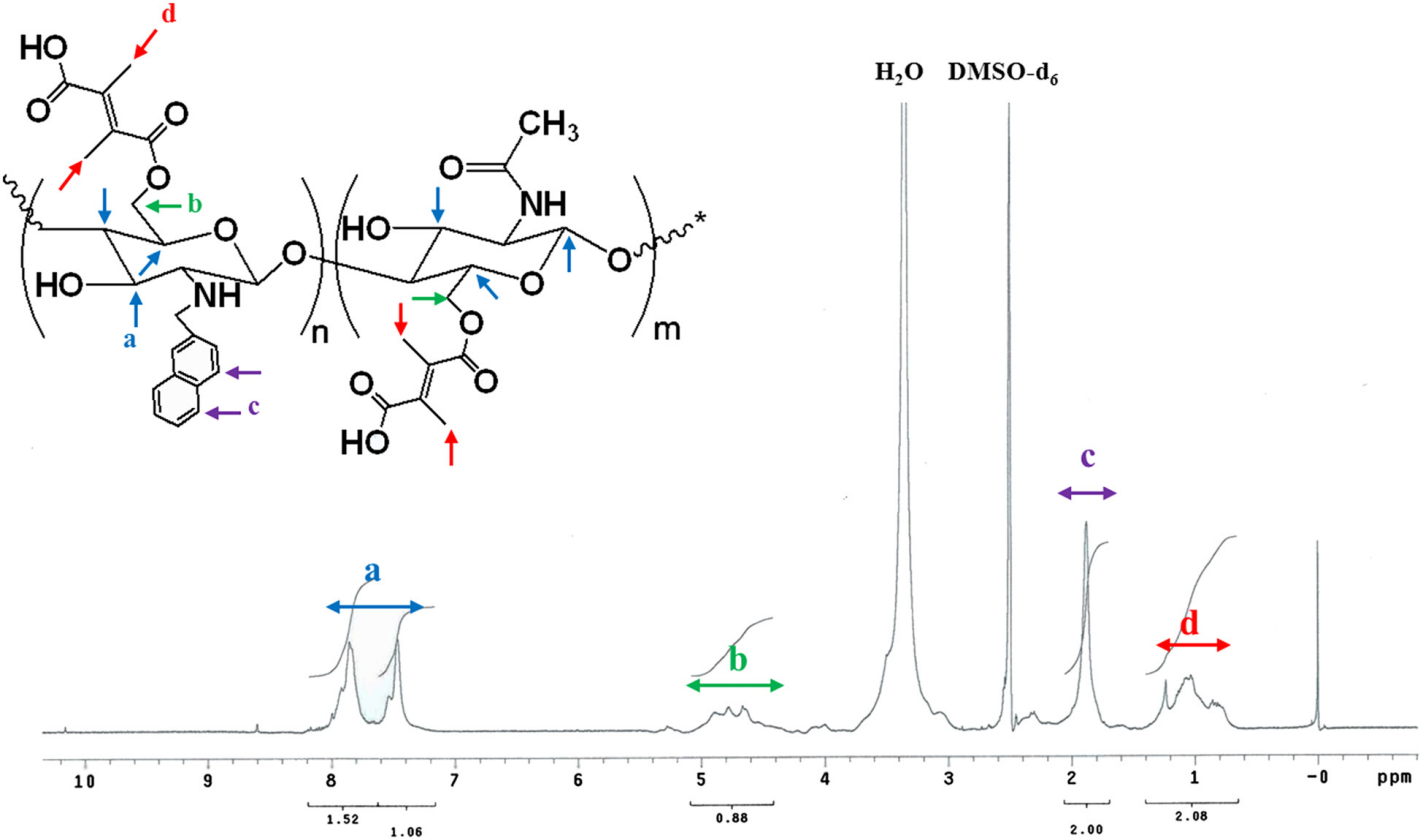
^**1**^**H-NMR spectrum of *N*-nap-*O*-MalCS. (a)** -CH- in aromatic ring. **(b)** -CH_2_-. **(c)** -CH. **(d)** -CH_3_.

**Figure 4:**
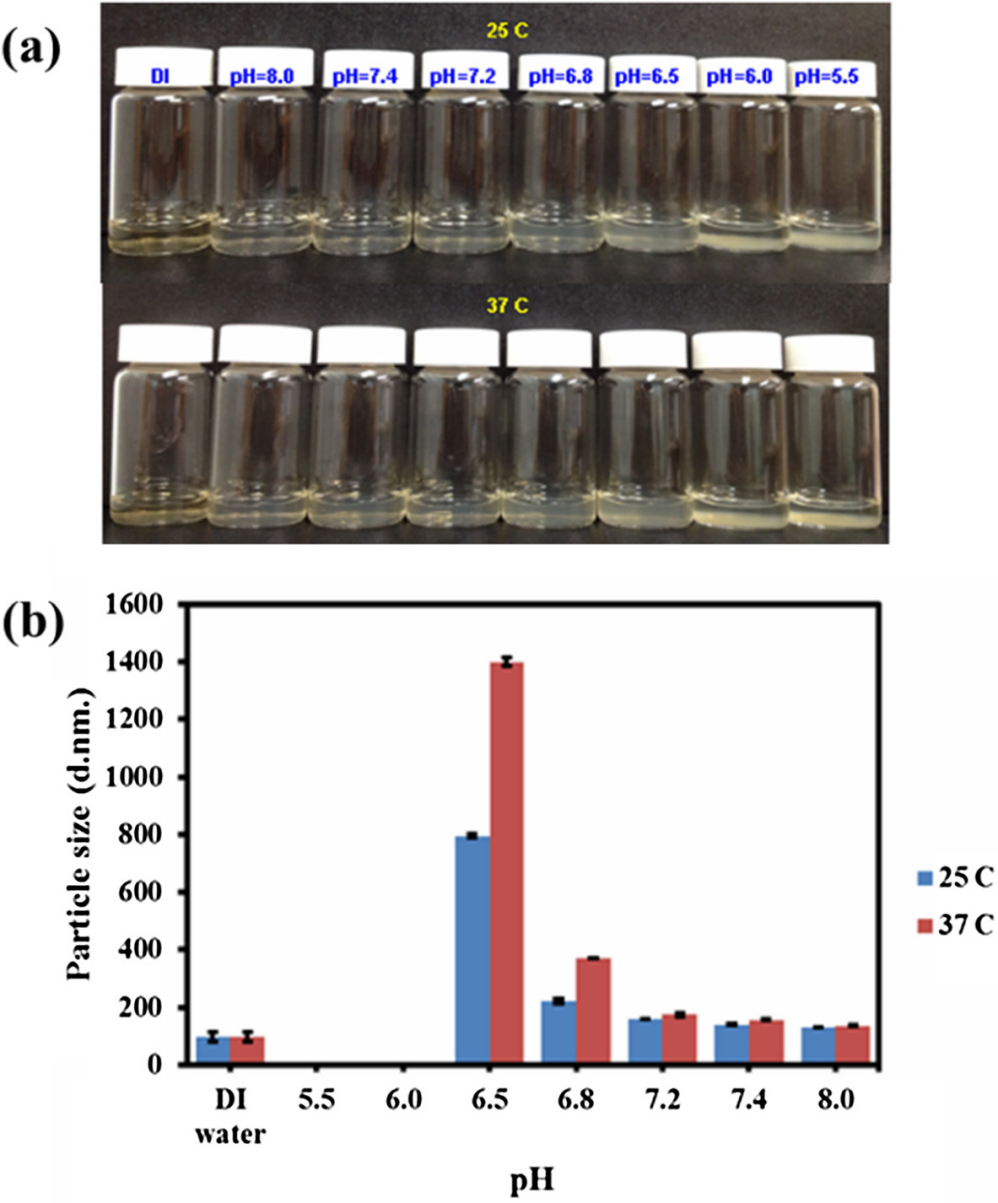
**Effect of *N*-nap-*O*-MalCS polymeric micelles in various pH conditions and temperatures. (a)** Stability. **(b)** Particle size.

*N*-naphthyl-*O*-dimethymaleoyl chitosan was synthesized by modifying chitosan with naphthyl groups at amino groups to complement their solubility and introduce amphiphilic properties [79]. Chitosan was reacted with naphthaldehyde to obtain an imine (Schiff base), which is easily converted into an *N*-naphthyl derivate by reduction with sodium borohydride or sodium cyanoborohydride (Figure 2a). Afterward, *N*-NapCS was introduced into the hydroxyl groups of chitosan by maleoylation with dimethylmaleic anhydride in DMF/DMSO to obtain *N*-nap-*O*-MalCS (Figure 2b) [67,68]. This synthetic compound was characterized by a ^1^H-NMR spectrum, and satisfactory analysis data were obtained (Figure 3). *N*-nap-*O*-MalCS was used to form nanopolymeric micelles by dialysis in various pH solutions. They were less than 200 nm at pH 7.2 to 8.0 but rapidly increased in size as the acidity of solution increased (Figure 4). Their sizes could not be measured at pH 5.5 and 6.0 (Figure 4a) due to aggregation. This was a result of the weakened solubility of *N*-nap-*O*-MalCS in the aqueous phase caused by acid hydrolysis of its maleoyl groups [80,81]. This phenomenon accelerated at 37°C compared to 25°C (Figure 4b). *N*-Nap-*O*-MalCS has a potential as a drug carrier because it can self-assemble with pH-sensitive behavior [67,68,79,82].

### Characterization of *N*-naphthyl-*O*-dimethymaleoyl chitosan-based drug-loaded magnetic nanoparticles

**Figure 5:**
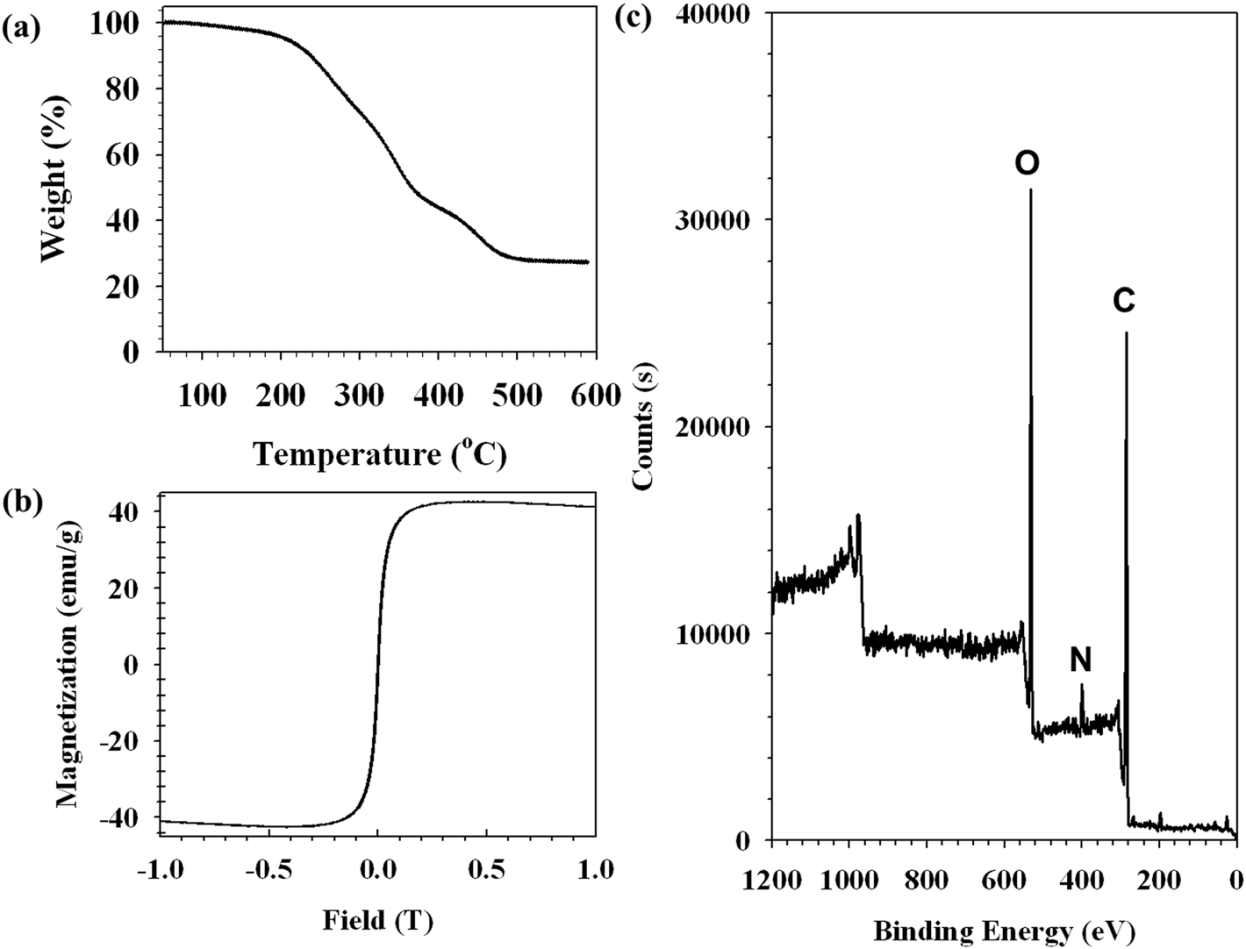
**Characterizations of *N*Chitosan-DMNPs. (a)** Thermogravimetric analysis (TGA), **(b)** magnetic hysteresis loops, and **(c)** XPS patterns of *N*-naphtyl-*O*-dimethymaleoyl chitosan-based drug-loaded magnetic nanoparticles (*N*Chitosan-DMNPs).

**Figure 6:**
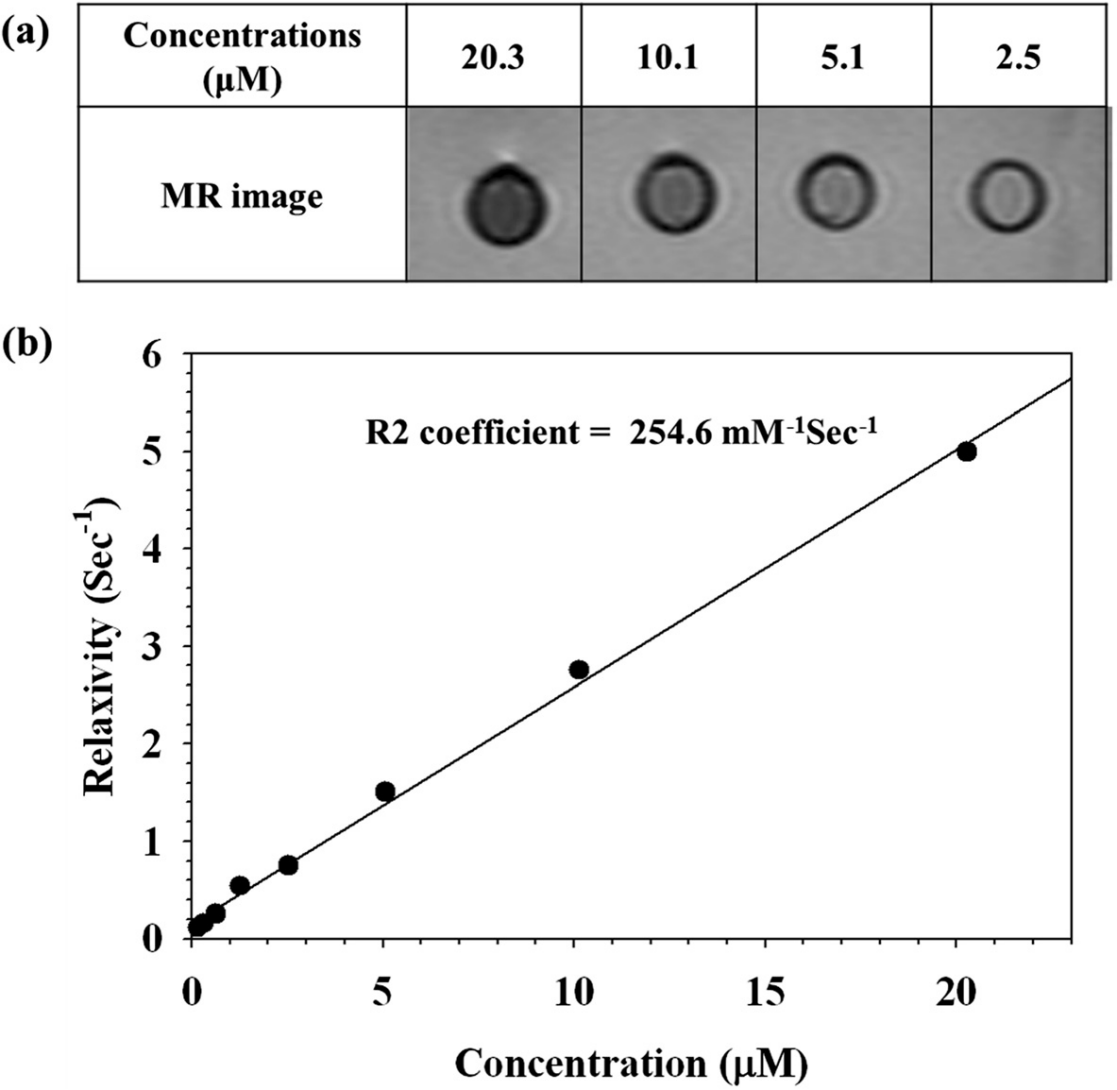
**Assessment of the ability of *N*Chitosan-DMNPs as MRI contrast agents. (a)***T*2-weighted MR images of *N*Chitosan-DMNPs in aqueous solution and **(b)** relaxation rate (*R*2) versus *N*Chitosan-DMNPs concentration.

NChitosan-DMNPs were prepared by a nanoemulsion method, in which naphtyl groups were absorbed on the hydrophobic surface of MNCs and DOX mainly caused by *van der Waals* force, and both their oxygen atoms and water molecules were interacted by hydrogen bonding. This interaction could lead to formation of *N*Chitosan-DMNPs dispersed in aqueous phase with high colloidal stability. *N*Chitosan-DMNPs were loaded with 27.5 wt.% MNCs and exhibited superparamagnetic behavior with a magnetization saturation value of 40.4 emu/g_Fe + Mn_ at 1.2 T (Figure 5). In addition, iron (Fe) and manganese (Mn) were not detected by X-ray photoelectron spectroscopy (XPS) analysis, which indicates that MNCs were safely encapsulated inside the *N*Chitosan-DMNPs (Figure 5). The availability of *N*Chitosan-DMNPs as MRI contrast agents was evaluated by measuring spin-spin relaxation times (*T*2) of water protons in the aqueous solutions using 1.5-T MR images. As the concentration of MNCs (Fe + Mn) in *N*Chitosan-DMNPs increased, the MR image was proportionally darkened with an *R*2 coefficient of 254.6/mMs, demonstrating that *N*Chitosan-DMNPs have sufficient ability as MRI contrast agents (Figure 6).

### pH-sensitive drug release properties

**Figure 7:**
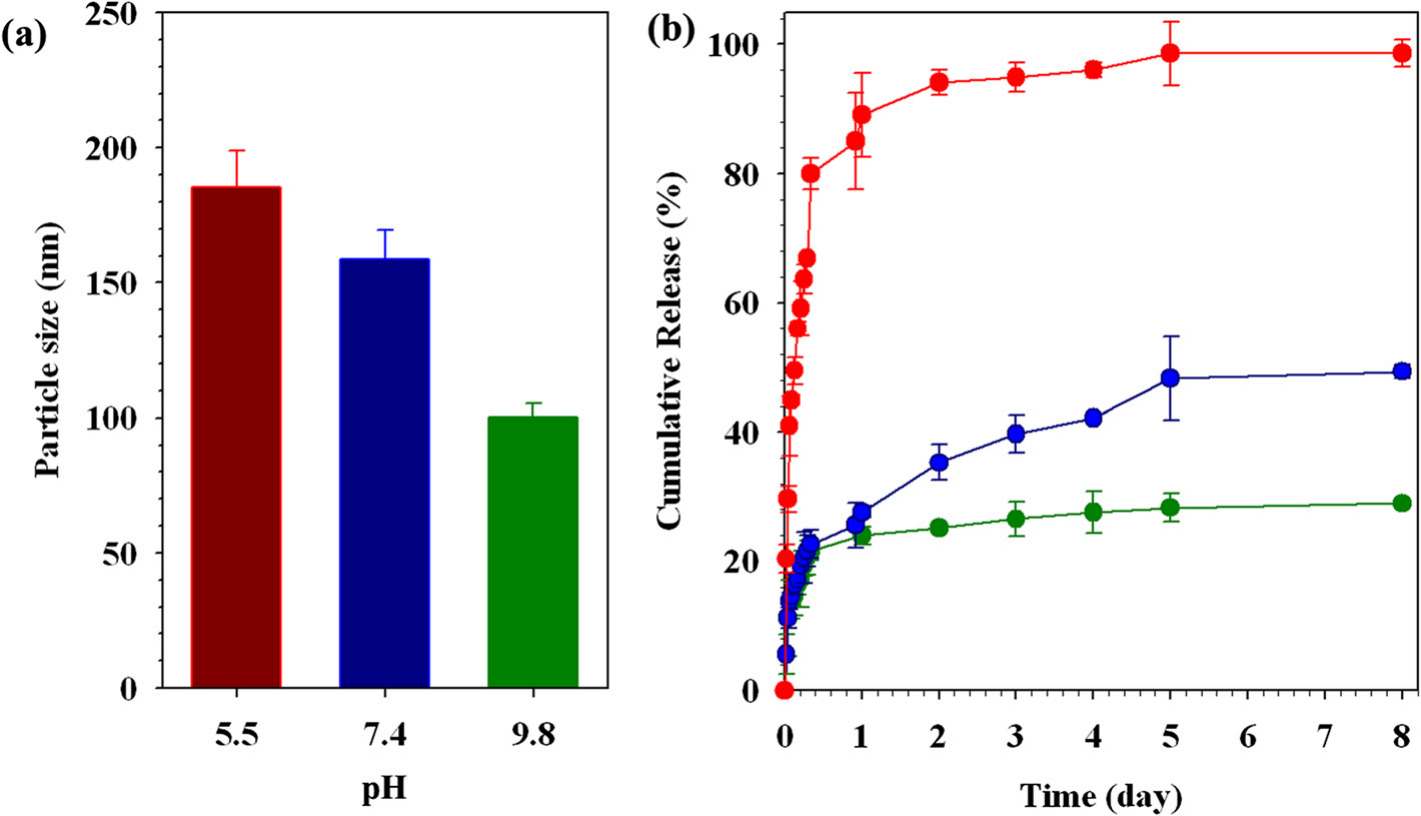
**Particle size of *N*Chitosan-DMNPs in different pH conditions (a) and pH-sensitive drug release profiles (b).** Red pH 5.5, blue pH 7.4, and green pH 9.8.

To investigate the pH-dependent behavior of *N*Chitosan-DMNPs, they were dispersed in different pH solutions (pH 5.5, 7.4, and 9.8) and their sizes were analyzed using laser scattering. *N*Chitosan-DMNPs in a pH 9.8 solution showed stable particle size around 100 nm (100.3 ± 4.9 nm), but their sizes increased slightly with increased buffer solution acidity (pH 5.5, 185.3 ± 13.5 nm and pH 7.4, 158.8 ± 10.6 nm) (Figure 7a) [17,20,30,83,84]. This is because the solubility of *N*-nap-*O*-MalCS of *N*Chitosan-DMNPs was weakened by acid hydrolysis of maleoyl groups, as mentioned above. This pH-dependent behavior was expected to induce pH-sensitive drug release profiles. DOX was abruptly released from *N*Chitosan-DMNPs under acidic conditions (pH 5.5) with about 90% of drug release within 24 h (Figure 7b), whereas only 20% of DOX was released at higher pH conditions (pH 7.4 and 9.8) during the same time period and both release profiles showed sustained release patterns for 8 days. This result implies that drugs could be released more from *N*Chitosan-DMNPs in acidic tumor sites than in normal tissues with decreased drug loss during blood circulation. After *N*Chitosan-DMNPs internalization by endocytosis, drug release could be further accelerated inside the acidic endosomes of tumor cells.

### Cellular uptake and cytotoxicity

**Figure 8:**
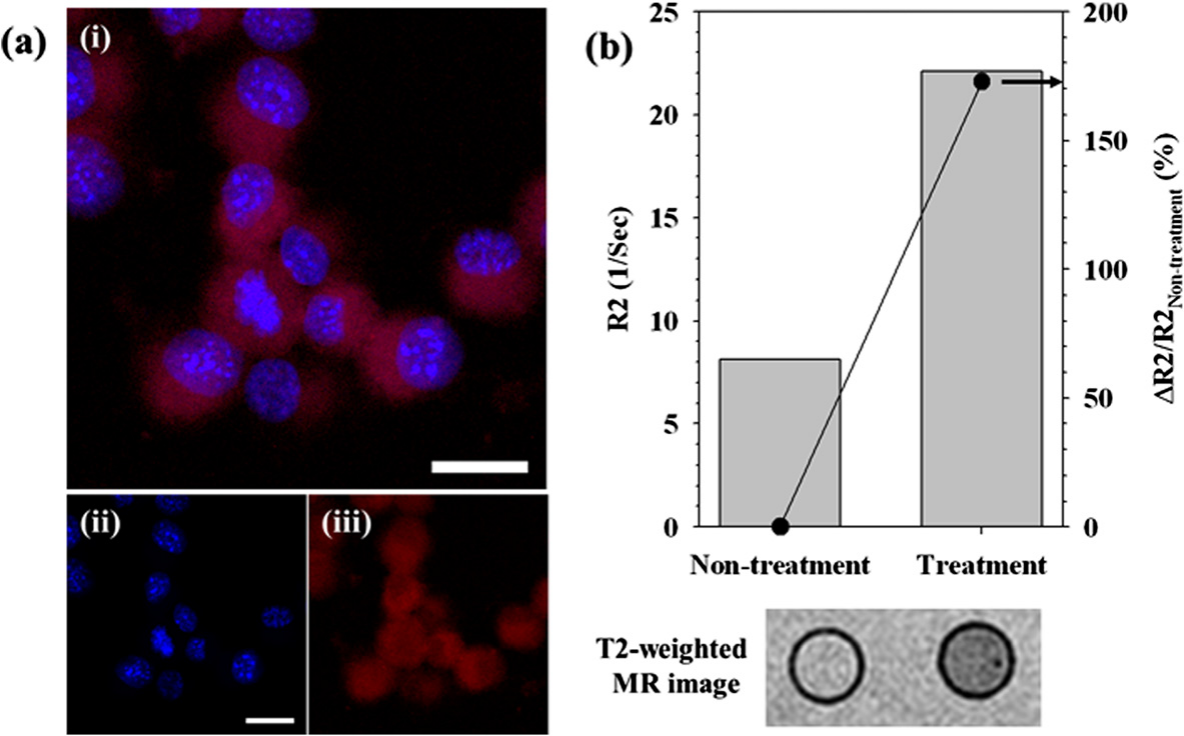
**Cellular internalization efficacy of *N*Chitosan-DMNPs. (a)** Fluorescence image of *N*Chitosan-DMNP-treated cells (i, merged image; ii, blue filter for Hoechst; iii, red filter for DOX). **(b)***T*2-weighted MR image and graph of △*R*2/*R*2 non-treatment for *N*Chitosan-DMNP-treated cells. Scale bars 50 μm.

**Figure 9:**
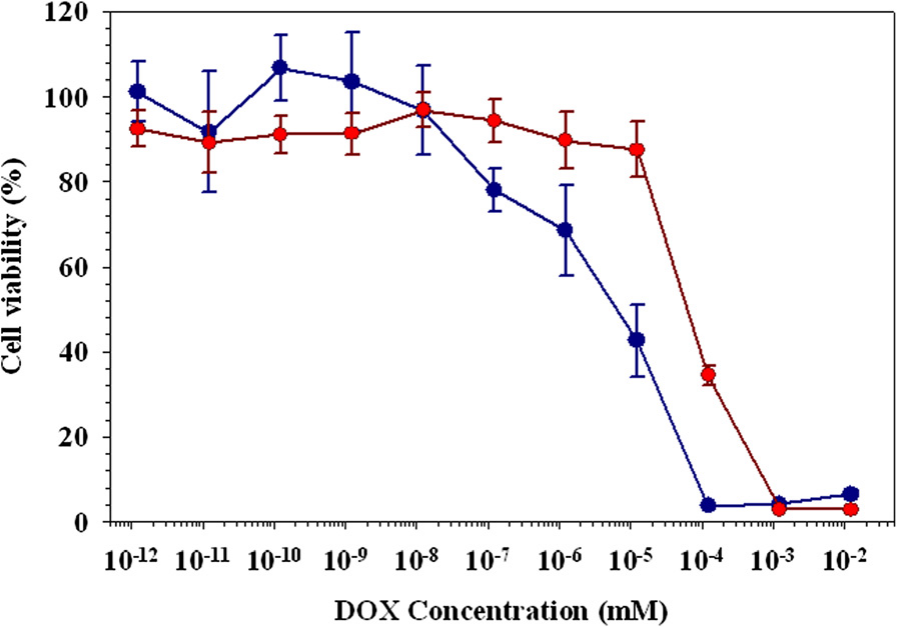
**Cell viability test of cells treated with DOX and *N*** **Chitosan-DMNPs (red, *N*** **Chitosan-DMNPs; blue, DOX).**

NIH3T6.7 cells were treated with *N*Chitosan-DMNPs and observed by confocal laser fluorescence microscopy to confirm their cellular uptake. Blue fluorescence indicated cell nuclei by Hoechst stains and red fluorescent signals are derived from cell nuclei and DOX. In Figure 8a, red fluorescence was generally observed in the intracellular regions, indicating released DOX from internalized *N*Chitosan-DMNPs. NIH3T6.7 cells incubated with *N*Chitosan-DMNPs also showed MR contrast effects compared to non-treated cells (non-treatment) (Figure 8b). The MR signal of NIH3T6.7 cells treated with *N*Chitosan-DMNPs was about 1.72-fold higher than that of non-treated cells, with an *R*2 value of 22.1/s (*R*2 value of non-treated cells: 8.10/s). The cytotoxicity of *N*Chitosan-DMNPs against NIH3T6.7 cells was evaluated by MTT assay (Figure 9) [85-87]. DOX-treated cells were also evaluated under the same conditions as a control.

DOX and *N*Chitosan-DMNPs exhibited dose-dependent cytotoxic effects on NIH3T6.7. DOX showed a higher cytotoxicity than *N*Chitosan-DMNPs because *N*Chitosan-DMNPs released DOX after their cellular internalization, while free DOX directly diffused and penetrated through cell membranes due to its low molecular weight.

### In vivo *theranostic effects of* N*Chitosan-DMNPs*

**Figure 10:**
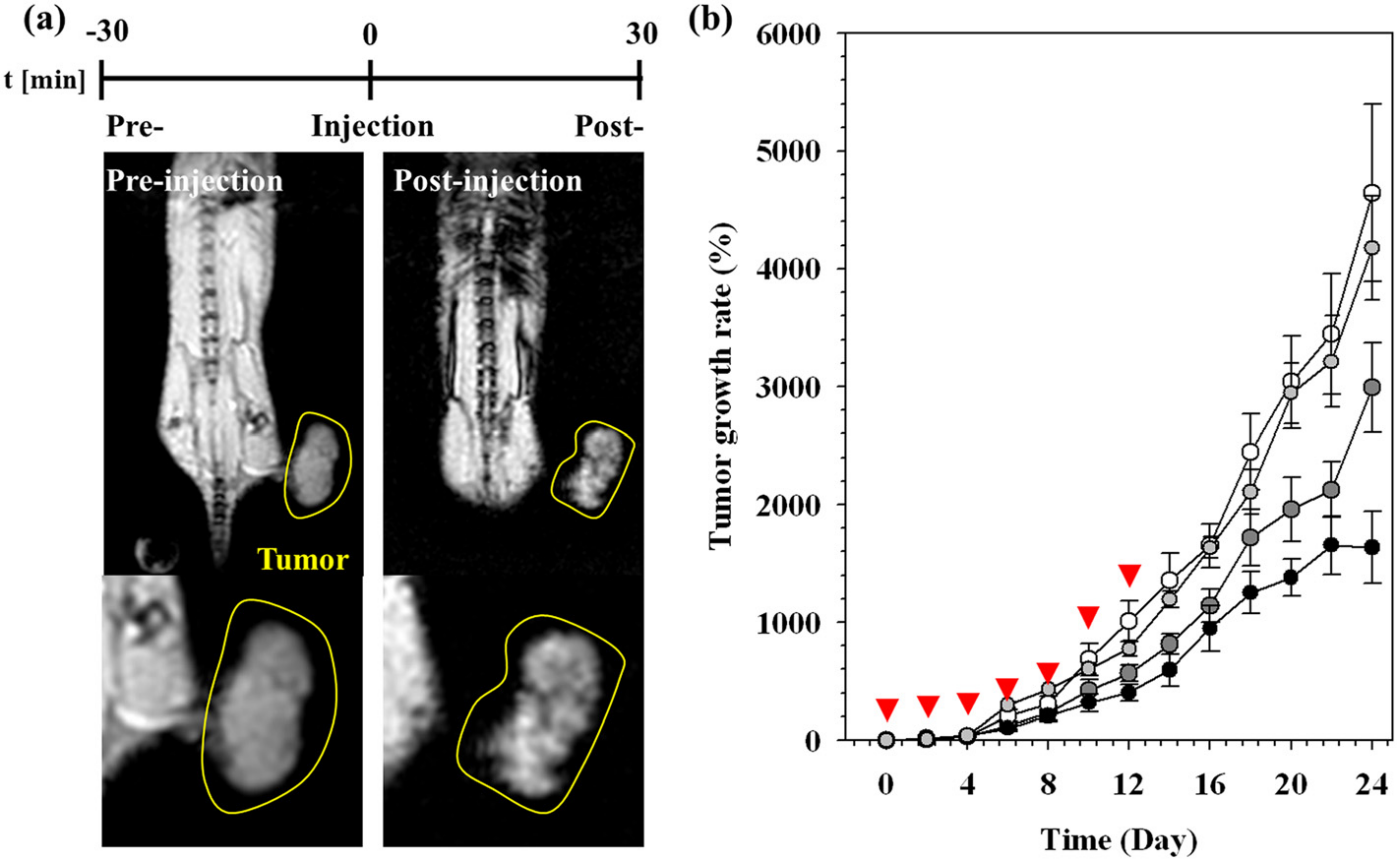
**MR imaging to assess intratumoral distributions of *N*Chitosan-DMNPs in tumor-bearing mice and comparative therapeutic efficacy. (a)***T*2-weighted MR images of tumor-bearing mice after intravenous injection of *N*Chitosan-DMNPs. Tumor regions are indicated with a yellow line boundary. **(b)** Comparative therapeutic efficacy study in the *in vivo* model (black, *N*Chitosan-DMNPs; gray, DOX; white, saline). Red arrowheads indicate the therapeutic dosing schedule of each therapeutic condition (*N*Chitosan-DMNPs, DOX, and saline).

The theranostic effects of *N*Chitosan-DMNPs were confirmed against an *in vivo* model [9,88,89]. To determine the therapeutic dosing schedule, intratumoral distributions of *N*Chitosan-DMNPs in tumor-bearing mice were investigated through MR images after intravenous injection into mouse tail veins (150 μg Fe + Mn, 3 mg/kg DOX). After injecting *N*Chitosan-DMNPs (post-injection), the black color gradually spread out in *T*2-weighted MR images following the peripheral blood vessels of the tumor area. This resulted from diffusion and permeation to tumor tissues across corresponding vascular distributions by an EPR effect (Figure 10a). The therapeutic dosing of *N*Chitosan-DMNPs were determined because these were maximally delivered within 1 h at the tumor sites and then over 80% of drug was released in the in the acidic environments within the tumor for 24 h, as judged from *in vivo* MRI and drug release profiling studies. Considering these results, we determined 2 days periodically to consistently maintain drug concentration within tumors for effective cancer therapy. NChitosan-DMNPs, free DOX, and saline were administrated to each subgroup of tumor-bearing mice via intravenous (i.v.) injection every 2 days for 12 days (injection on days 0, 2, 4, 6, 8, 10, and 12). Tumor sizes were monitored for 24 days. *N*Chitosan-DMNPs exhibited significant tumor growth inhibition with an average tumor growth rate of 1,638.1 ± 306.9% compared to the control (free DOX and saline) groups (saline, 4,642.8%; free DOX, 2,991.9%) (Figure 10b). Although *N*Chitosan-DMNPs could not completely suppress tumor growth, tumor growth inhibition was more effective than with saline or free DOX. During the experimental period, no loss in mice body weight was observed.

## Conclusions

We have formulated theranostic nanocomposites, *N*Chitosan-DMNPs, based on *N*-nap-*O*-MalCS for effective cancer therapy. *N*Chitosan-DMNPs exhibited a pH-sensitive drug release pattern with MR imaging due to the pH-sensitive properties of *N*-nap-*O*-MalCS. Furthermore, theragnostic efficacies of *N*Chitosan-DMNPs were confirmed in the *in vivo* model by determining their therapeutic dosing schedule based on drug release profiling and *in vivo* MRI study. From these results, *N*Chitosan-DMNPs are expected to play a significant role in the dawning era of personalized medicine.

## Abbreviations

DMNPs: Drug-loaded magnetic nanoparticles; DOX: Doxorubicin; MNCs: Magnetic nanocrystals; MRI: Magnetic resonance imaging.

## Competing interests

The authors declare that they have no competing interests.

## Authors’ contributions

EKL and WS performed the experiments, suggested the scheme, and wrote the manuscript. YC and EJ performed the experiments. HL, BK, and EK reviewed the scheme and contents. SH and JSS revised the manuscript critically for important intellectual content. SJC and YMH supervised the project. All authors read and approved the final manuscript.
